# Optimization of salt concentration in PEG-based crystallization solutions

**DOI:** 10.1107/S0909049510035995

**Published:** 2010-11-05

**Authors:** Mari Yamanaka, Koji Inaka, Naoki Furubayashi, Masaaki Matsushima, Sachiko Takahashi, Hiroaki Tanaka, Satoshi Sano, Masaru Sato, Tomoyuki Kobayashi, Tetsuo Tanaka

**Affiliations:** a Confocal Science Inc., Japan; b Maruwa Foods and Biosciences Inc., Japan; c Aino Gakuin College, Japan; d Japan Aerospace Exploration Agency, Japan

**Keywords:** protein crystallization, optimization, salt concentration, polyethylene glycol, ionic strength

## Abstract

Although polyethylene glycol (PEG) is the most widely used precipitant in protein crystallization, the concentration of co-existing salt in the solution has not been well discussed. To determine the optimum salt concentration range, several kinds of protein were crystallized in a 30% PEG 4000 solution at various NaCl concentrations with various pH levels. It was found that, if crystallization occurred, the lowest effective salt concentration depended on the pH of the protein solution and the pI of the protein molecule; that is, higher salt concentrations were required for crystal growth if the difference between pH and pI was increasing. The linear relationship between the charge density of the protein and the ionic strength of the crystallization solution was further verified. These results suggested that the lowest effective concentration of salt in a crystallization solution can be predicted before performing a crystallization experiment. Our results can be a tip for tuning crystallization conditions by the vapor-diffusion method.

## Abstract 2

Optimal salt concentration in a PEG-based crystallization solution is important for successful crystal growth and can be predicted prior to performing crystallization experiments.

## 1. Introduction   

The success rate of screening for suitable protein crystallization conditions is often low owing to the extensive number of variables that can be altered, such as the amount and types of salt, buffer, pH, precipitants and other chemical components (Cudney *et al.*, 1994 ▶). Based on the review by Chayen & Saridakis (2002 ▶), from cloned protein to structure determination the largest failure rate is in obtaining good crystals, but little attention has been given to improving methods of optimization of crystallization conditions.

Polyethylene glycol (PEG) is a frequently used precipitant reagent in protein crystallization solutions. Bonneté (2007 ▶) reported that the concentration of salt was limited to roughly 300 m*M* when salt and polymer were both used and that there were some synergetic effects between polymer and salt. It was considered that low salt concentrations screened the macromolecular charges and decreased the electrostatic repulsive force between the molecules. However, the salt concentration required to screen and to grow a crystal has not been studied yet.

In this report we show the results of experiments to determine the concentration range of salt in PEG solutions at several pH levels which can be used to grow crystals, and discuss the results from the charge density viewpoint, proposed by Matsushima & Inaka (2007 ▶). We found that there is a good linear relationship between the charge density of the macromolecule and the ionic strength of the reservoir solution.

## 2. Materials and methods   

### 2.1. Crystallization   

A counter-diffusion method (García-Ruiz & Moreno, 1994 ▶; Otálora *et al.*, 2009 ▶) was used here because it could control better the concentrations of the chemicals in the solution by a simpler diffusive process than the vapor-diffusion method, although the vapor-diffusion method is widely used for protein crystallization by most crystallographers. We use a gel-tube method (Tanaka *et al.*, 2004 ▶), which is a modification of the original capillary counter-diffusion method of García-Ruiz & Moreno (1994 ▶). Assembly of the crystallization device has been described previously (Tanaka *et al.*, 2004 ▶). Briefly, a 0.3 mm-diameter capillary was filled with protein solution to a length of 30 mm (2.1 µl) and its upper end was sealed with clay before being plugged with a silicone tube filled with agarose gel, the length of which was 5 mm. The capillary was placed into the test tube in which 3 ml of reservoir solution was loaded. The gel allowed components of the protein and reservoir solutions to diffuse through each other. Agarose gel in the tube was pre-equilibrated with respective reservoir solutions. The crystallization was performed at 293 K for two weeks and checked on days 1, 2, 3, 7 and 14 by microscope. At least two capillaries were used for respective crystallization conditions.

### 2.2. Proteins   

The proteins, hen egg-white lysozyme (Seikagaku), α-amylase derived from *Aspergillus oryzae* (Shinnihon Chemicals) and glucose isomerase (Hampton Research), were chosen based on availability and crystallizability. The proteins were further purified: lysozyme by CM-TOYOPEARL (TOSO), α-amylase and glucose isomerase by Q Sepharose HP (GE Healthcare). The purified proteins showed a single band through SDS-PAGE and native-PAGE. Finally, 30 mg ml^−1^ lysozyme in 50 m*M* acetate buffer pH 4.5, 30 mg ml^−1^ α-amylase in 50 m*M* Tris-HCl pH 7.5 and 20 mg ml^−1^ glucose isomerase in 20 m*M* Tris-HCl and 200 m*M* NaCl pH 7.5 were prepared.

### 2.3. Reservoirs   

Several series of reservoir solutions, which were a mixture of 30% PEG 4000 as a precipitant, NaCl of 0 m*M* to 700 m*M* as a salt, and several kinds of buffers including 50 m*M* acetate buffer at pH 4.5 and 5.5, 50 m*M* HEPES-NaOH at pH 7.0 and 50 m*M* Tris-HCl at pH 9.0, were prepared (Table 1 ▶).

### 2.4. Calculation   

To determine the concentration profile of NaCl and PEG 4000 in the capillary tubing and the gel, the concentration change was calculated by one-dimensional simulation (Tanaka *et al.*, 2004 ▶) using diffusion constants of 1.2 × 10^−9^ m^2^ s^−1^ and 0.16 × 10^−9^ m^2^ s^−1^, respectively.

The ionic strengths of the reservoir solutions were calculated using p*K_a_* values of acetate, HEPES and Tris buffers of 4.80, 7.55 and 8.06, respectively, and the NaCl concentration in each solution. The pI of the proteins was calculated using p*K* values of amino acids derived from the report of Sillero & Maldonado (2006 ▶).

The charge density, which is the amount of charge normalized to the protein molecular volume, was calculated using the following equation, which was proposed by Matsushima & Inaka (2007 ▶),
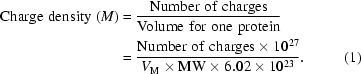
The number of charges is the net amount of charge of one protein molecule at a certain pH. It is calculated using the amino acid composition and the p*K* values of amino acids. *V*_M_ is Matthew’s coefficient which is already deposited in the Protein Data Bank. MW is the molecular weight of the protein molecule calculated using the amino acid composition.

## 3. Results   

The crystallization results are summarized in Table 2 ▶. The appearance of crystals, oil and precipitate observed at day 14 are indicated. More than two capillaries were used and crystallization was observed reproducibly for each crystallization condition.

In the experiments with lysozyme with buffer solution pH 4.5, protein solutions in the capillaries were still clear even at day 14 with 0 m*M* to 300 m*M* NaCl. With 400, 500, 600 and 700 m*M* NaCl, crystals were observed at days 14, 7, 3 and 2, and they grew at the position 11–30, 8–12, 5–25 and 4–30 mm, respectively, from the gel-tube site of the capillaries at day 14.

**Figure 1:**
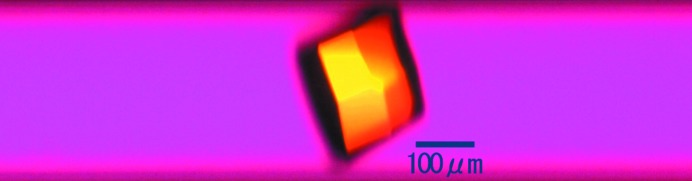
Crystal of lysozyme. A crystal was obtained in 50 m*M* HEPES pH 7.0 with 30% PEG 4000 and 700 m*M* NaCl. It was observed 7 days after the sample loading.

For lysozyme with buffer solution pH 7.0, protein solutions in the capillaries were still clear even at day 14 with 0 m*M* to 200 m*M* NaCl. With 300, 400, 500, 600 and 700 m*M* NaCl, crystals were observed at days 14, 14, 7, 7 and 7, and they grew at the position 20, 8–26, 5–18, 0–30 and 17–30 mm, respectively, from the gel-tube site of the capillaries at day 14 (Fig. 1 ▶).

**Figure 2:**
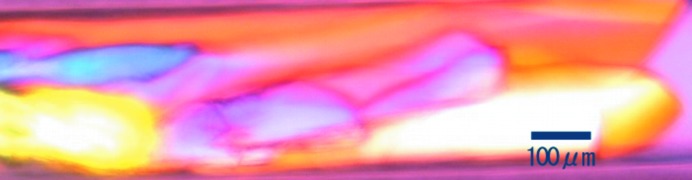
Crystal of α-amylase. A cluster of rod-shaped crystals of α-amylase was obtained in 50 m*M* acetate buffer pH 5.5 with 30% PEG 4000 and 300 m*M* NaCl. It was observed 7 days after the sample loading.

For α-amylase with buffer solution pH 5.5, protein solution in the capillaries was clear even at day 14 with 0 m*M* NaCl. A cluster of rod-shaped crystals appeared at day 1 with 100 m*M* and 200 m*M* NaCl. With 300 m*M* NaCl, a cluster of rod-shaped crystals appeared in one capillary (Fig. 2 ▶) and oil was observed in the other capillary at day 1. With 400 m*M* NaCl, oil appeared at day 1. In those capillaries with 300 m*M* or 400 m*M* NaCl in which oil was observed, a cluster of rod-shaped crystals appeared at day 7. At day 14, the clusters of crystals were observed at the position 0–5 mm from the gel-tube site of all the capillaries with 100, 200 and 300 m*M* NaCl. With 400 m*M* NaCl, crystals and oil were observed at the same position.

For α-amylase with pH 7.0, the protein solution was clear with 0 m*M* and 100 m*M* NaCl even at day 14. Oil appeared at day 1 with 200 m*M* to 700 m*M* NaCl. After the appearance of oil, at day 3, a cluster of rod-shaped crystals appeared with 200 m*M* and 300 m*M* NaCl. At day 14, the crystals and/or oil were observed at the position 0–2, 0–4, 0–8, 0–8, 0–9 and 0–9 mm from the gel-tube site of the capillaries with 200, 300, 400, 500, 600 and 700 m*M* NaCl, respectively.

For α-amylase with pH 9.0, the protein solution was clear with 0 m*M* and 100 m*M* NaCl even at day 14. Oil appeared at day 1 with 200 m*M* to 700 m*M* NaCl without crystals until day 14 at the position 0–5 mm from the gel-tube site of all the capillaries with 200, 300, 400, 500, 600 and 700 m*M* NaCl.

**Figure 3:**
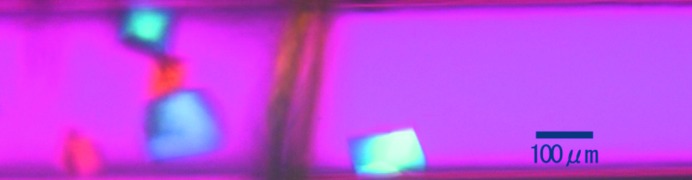
Crystal of glucose isomerase. Crystals of glucose isomerase were obtained in 50 m*M* Tris-HCl buffer pH 9.0 with 30% PEG 4000 and 500 m*M* NaCl. They were observed 1 day after the sample loading.

With glucose isomerase at pH 7.0 and pH 9.0, the protein solution was clear in the solution with 0 m*M* and 100 m*M* NaCl even at day 14. But many small crystals appeared at day 1 in all of the capillaries (Fig. 3 ▶) with 200 m*M* to 700 m*M* NaCl. All of them were accompanied by precipitate except for 200 m*M* NaCl at pH 9.0. At day 14, the crystals were observed at the position 0–2 mm from the gel-tube site of the capillaries with 200 m*M* NaCl at pH 9.0. The crystals and precipitate were observed at the position 0–9, 0–12, 0–18, 0–15, 0–16 and 0–15 mm from the gel-tube site of the capillaries with 200, 300, 400, 500, 600 and 700 m*M* NaCl, respectively, at pH 7.0 and at the position 0–3, 0–6, 0–5, 0–5 and 0–5 mm from the gel-tube site of the capillaries with 300, 400, 500, 600 and 700 m*M* NaCl, respectively, at pH 9.0.

In every case any change emerged between day 14 and two months.

**Figure 4:**
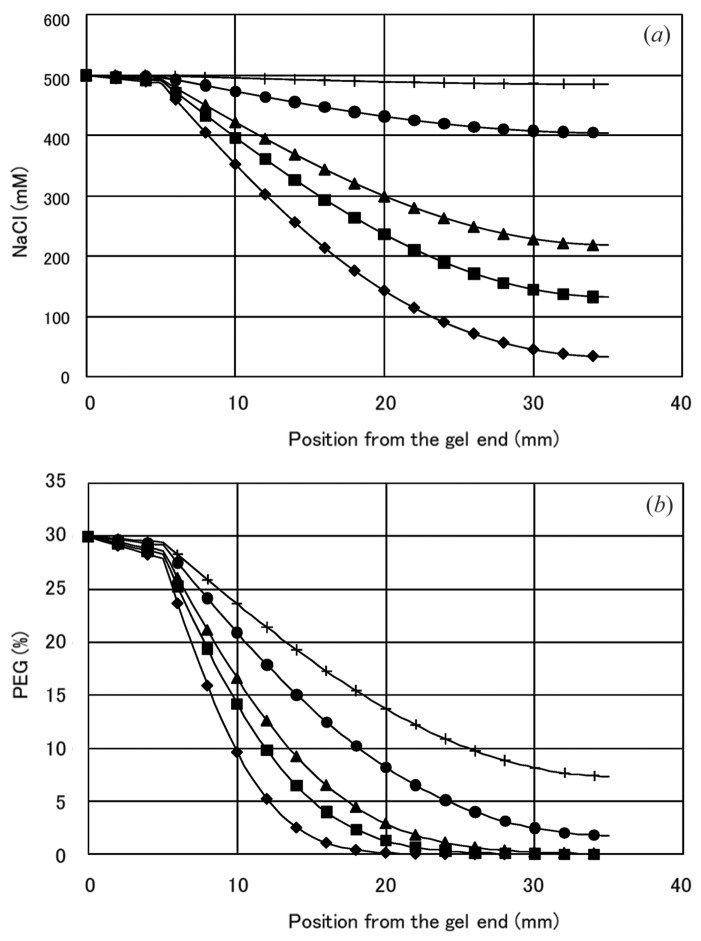
Diffusion profile in a capillary. The results of one-dimensional simulation of the diffusion of NaCl (*a*) and PEG 4000 (*b*) in the capillary are shown for 500 m*M* NaCl and 30% PEG 4000 as a reservoir solution. The concentrations of these components along the capillary tubing and the gel part are shown. Diamonds: day 1; squares, day 2; triangles, day 3; circles, day 7; plus signs, day 14.

The results of the one-dimensional simulation of NaCl and PEG 4000 concentration profiles in the capillary are shown in Fig. 4 ▶ for the experiment with 500 m*M* NaCl and 30% PEG 4000 as the reservoir solution. The concentrations of these components along the capillary tubing and the gel part at day 1, 2, 3, 7 and 14 after a solution loading are shown. Although the concentration of PEG 4000 does not reach an equilibrium, that of NaCl almost reaches the concentration in the reservoir at day 14.

The ionic strengths of the marginal solutions and the charge densities of the proteins were calculated and shown in Tables 1 ▶ and 3 ▶.

## 4. Discussion   

Since the NaCl concentration mostly reached equilibrium in the capillaries through the counter-diffusion method before day 14 and no change emerged in the capillaries after two months, we can discuss the effect of salt concentration on crystallization. It is commonly said that there is some marginal NaCl concentration for the emergence of crystals, oil or precipitate. If the concentration is lower, neither crystals, oil nor precipitate would appear. There may be some tendency to obtain oil or precipitate if the concentration of NaCl is higher.

**Figure 5:**
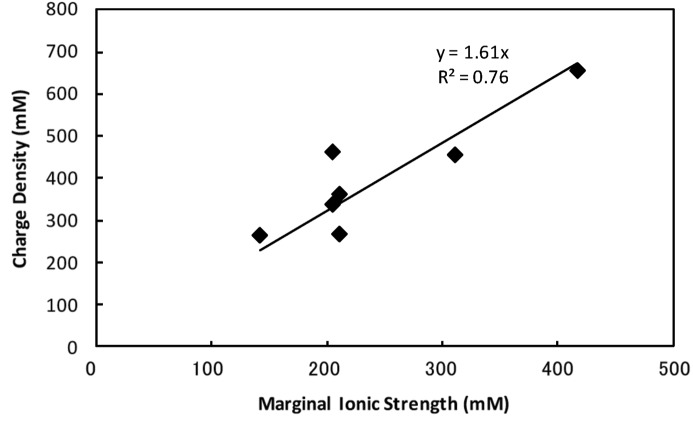
The relationship between the marginal ionic strength of the solution and the charge density of the protein. The coefficient of the linear relationship is 1.61 (*R* ^2^ = 0.76).

According to Bonneté (2007 ▶), the marginal concentration of NaCl may have some relation to the electrostatic screen effect. To estimate this effect we used the ionic strength of NaCl at the lowest concentration when crystals were observed (Table 3 ▶). From our calculation the marginal ionic strength increases when the difference between the pH of the solution and the pI of the proteins is large, which is consistent with the idea of the electrostatic screen effect of a salt. Fig. 5 ▶ shows the protein charge density values plotted against the marginal ionic strengths. A clear linear relationship, the coefficient of which was 1.61 (*R* ^2^ = 0.76), was found. Using this relationship the lowest concentration of the salt in the PEG 4000 solution can be predicted prior to performing crystallization experiments, although the *V*_M_ value is required. Kantardjieff & Rupp (2003 ▶) reported a plausible *V*_M_ value for various proteins, by which we can also predict the salt concentration of a protein which has not yet been crystallized.

Our results can also provide a tip for using the vapor-diffusion method. One of the differences between counter-diffusion and vapor-diffusion is the concentration change of salt in a crystallization drop. In the vapor-diffusion method a protein solution and a reservoir solution are usually mixed in a drop at a 1:1 ratio. Then crystallization occurs in the drop in which the components are concentrated through water loss. If the original protein and reservoir solution do not have enough salt, the concentration in the drop does not reach the marginal concentration level. If the original protein and reservoir solution has a significant amount of salt, the concentration in the drop easily becomes higher than that in the reservoir solution. Therefore, in the vapor-diffusion method, keeping the salt concentrations neither too low nor too high in the protein solution is important for successful crystallization. In other words, an unsuccessful PEG-based crystallization condition can be changed to a successful one if the salt concentration is well optimized.

## Figures and Tables

**Table 1:** Components and ionic strength of each reservoir solution Reservoir solutions with four buffers and eight NaCl concentrations were used. The ionic strengths of the solutions were calculated with p*K_a_* values of acetate, HEPES and Tris of 4.80, 7.55 and 8.06, respectively.

pH	4.5	5.5	7.0	9.0
Buffer	50 m*M* acetate	50 m*M* acetate	50 m*M* HEPES-NaOH	50 m*M* Tris-HCl
Precipitant	30% PEG 4000			
NaCl	0, 100, 200, 300, 400, 500, 600, 700m*M*			
Ionic strength	17717m*M*	42742m*M*	11711m*M*	5705m*M*

**Table 2:** Results of crystallization Conditions in which crystals, oil and/or precipitate were observed are indicated by C, O or P, respectively.

		Lysozyme		-Amylase			Glucose isomerase	
pH		4.5	7.0	5.5	7.0	9.0	7.0	9.0
NaCl (m*M*)	0	Clear	Clear	Clear	Clear	Clear	Clear	Clear
	100	Clear	Clear	C	Clear	Clear	Clear	Clear
	200	Clear	Clear	C	C, O	O	C, P	C
	300	Clear	C	C	C, O	O	C, P	C, P
	400	C	C	C, O	O	O	C, P	C, P
	500	C	C	-	O	O	C, P	C, P
	600	C	C	-	O	O	C, P	C, P
	700	C	C	-	O	O	C, P	C, P

**Table 3:** Comparison of the marginal ionic strength and the calculated charge density The pI was calculated using p*K* values derived from the report of Sillero Maldonado (2006 ▶). The ionic strengths of the reservoir solution were calculated using p*K_a_* values of acetate, HEPES and Tris buffers as 4.80, 7.55 and 8.06, respectively, and the lowest NaCl concentration in each pH when crystals are observed. The charge density was calculated using equation (1) with *V*_M_ values shown in the table.

	Lysozyme		-Amylase			Glucose isomerase	
pH	4.5	7.0	5.5	7.0	9.0	7.0	9.0
Calculated pI	10.7		4.4			5.0	
*V*_M_/PDB code	2.08/1bwh		2.18/6taa			2.78/1xib	
Marginal ionic strength (m*M*)	417	311	142	211	205	211	205
Calculated charge density (m*M*)	654	455	265	362	462	268	338
